# Assessing the Impacts of Drought on Grassland Net Primary Production at the Global Scale

**DOI:** 10.1038/s41598-019-50584-4

**Published:** 2019-10-01

**Authors:** Qian Wang, Yue Yang, Yangyang Liu, Linjing Tong, Qi-peng Zhang, Jianlong Li

**Affiliations:** 1 Department of Ecology, School of Life Sciences, Nanjing University, Nanjing, 210093 China; 2 Nanjing Institutes of Environmental Sciences, Ministry of Environmental Protection of the People’s Republic of China, Nanjing, 210042 China; 3 School of Geographic Sciences, Nanjing Normal University, Nanjing, 210023 China

**Keywords:** Environmental sciences, Natural hazards

## Abstract

Quantitatively assessing the impacts of drought on grassland has significant implications to understand the degradation mechanism and prevention degraded grassland. In this study, we analyzed the relationship between grassland drought and grassland Net Primary Productivity (NPP) based on the self-calibrated Palmer Drought Severity Index (scPDSI) from 1982 to 2008. The results showed that the global grassland scPDSI value had a slightly increasing trend with the rate of 0.0119 per year (R^2^ = 0.195), indicating that the global grassland drought lighter to some extent during study period. Moreover, the correlation coefficient between annual grassland NPP and scPDSI was from −0.83 to 0.92. The grassland NPP decreased under mild drought from 1992 to 1996. Additionally, the correlation coefficient between scPDSI and NPP for each grassland type was: Closed Shrublands > Non-woody grassland > Savannas > Open Shrublands > Woody Savannas, indicating that drought had difference influences on the different grassland types. Our results might provide the underlying insights needed to be guide for the effects of extreme weather events on grassland NPP.

## Introduction

Grassland ecosystem, as the earth’s largest terrestrial ecosystem^1,2^, provides a large number of economic products and other ecological services^3^. Climate changes may impacts the fluxes of carbon, water and energy between the biosphere and the atmosphere^4^. Therefore, it is important to understand the response of grassland ecosystems to climate changes. To some extent, grassland ecosystem is also closely related to socio-economic development and regional ecological security^5^. Therefore, grassland ecosystem is of great significance to the sustainable development of human beings.

Grasslands cover approximately 40% of the ice-free global terrestrial surface^6,7^ and contain around 30% of global total soil carbon (C) stocks^8^. Since grassland plays an important role in ecosystem cycles, it is necessary to quantitatively evaluate grassland ecosystems^9–11^. Net primary production (NPP) is a measure of the net amount of carbon and plays an important role in the global carbon balance^12^, as well as in climate change^13^. As the foundation of energy flow and nutrient cycle for organisms, NPP is an organic compounds produced by photosynthesis.^13^. Thus, the disturbances of ecosystem structure and function might influences on terrestrial carbon cycle. There are extensive studies about the effects of major disturbances on the terrestrial carbon cycle, such as overgrazing^14^, urbanization, fire, and deforestation^15,16^. It is well known that climate change and its related extreme events have range crucial consequences markedly on global carbon balance^17,18^. Droughts are one of the major natural hazards, which can reduce plant productivity, lead to widespread plant death and restrict the geographical distribution of plant species^10,19–21^. Thus, droughts can be regard as one of the disturbances of ecosystem structure and function. Since frequency and intensity of droughts are supposed to increase in many regions in the 21st century^22^, it is expected to impact the carbon cycle more strongly in the future^23,24^. As one of the main contents of climate-vegetation researches, NPP change caused by global change has always been a research hotspot^25^. Therefore, a better understanding in spatio-temporal variations in NPP and its feedback on drought will improve the prediction of future terrestrial carbon flux^26^.

In recent years, a few efforts have been made to investigate the productivity of terrestrial ecosystems influenced by droughts^9^. The previous studies conducted the impact of drought on vegetation carbon storage at the difference time scales^27–30^. For example, some researchers conducted the net primary production distribution and response to drought at Regional-to-Local Scales^27^. A continental scale survey of the decline in primary productivity in Europe was conducted^31^. Furthermore, some study was conducted in arid and semi-arid regions^32^. Besides, the global-scale analysis of the carbon cycle sensitivity to drought also reported^33^. They found that the global NPP affected by droughts decreased in the Southern Hemisphere but increased in the Northern Hemisphere from 2000 to 2009. Most recent findings showed that how droughts impact NPP is Hotpoint issues. However, to our knowledge, the subsequent of droughts influences on terrestrial carbon cycle are not well explored^13,34^ at the global scale, especially from the aspect of the difference vegetation types. Although previous studies have focused on the impact of droughts on vegetation carbon storage, few have focused on the impacts of droughts on the difference vegetation types at the global scale. To address this, the specific objectives of present study were to discuss the impact of drought on grassland NPP at difference drought levels based on scPDSI during 1982 to 2008. We believed that this will improve an understanding of the impacts of impacts of extreme weather events on grassland ecosystem.

## Results

### Spatial and temporal characteristics of grassland drought from 1982 to 2008

The overall characteristics of the annual global grassland scPDSI

**Figure 1:**
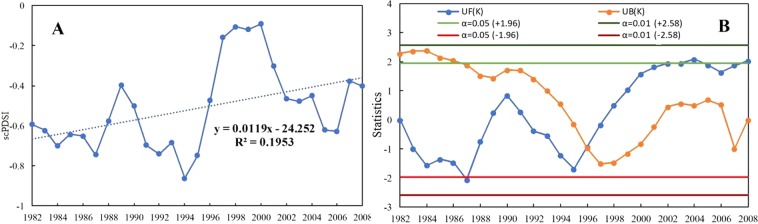
The overall characteristics of the annual global grassland scPDSI and its MK test during 1982 to 2008. (**A**) is the overall change trend of the nnual global grassland scPDSI from 1982 to 2008. (**B**) shows the MK test of the annual global grassland scPDSI from 1982 to 2008 (UF(k),and UB(k) curve is marked by the blue line and yellow blue line, respectively; and the four straight line is the threshold limit line).

Figure 1A shows the annual average changes of grassland scPDSI from 1982 to 2008. We can see that global grassland scPDSI value showed a slightly increasing trend with the rate of 0.0119 per year (R^2^ = 0.195) during study period, indicating that the global grassland drought degree was alleviated during the study period.

Figure 1B shows the MK test of the annual global grassland scPDSI during study period. The result shows that the annual global grassland scPDSI presented a slight fluctuating trend. The UF(k) showed a decreasing trend during 1982–1988, and an increasing trend from 1989 to 1991, and then a downward trend during 1992–1997, and an increasing trend after 1998. Additionally, the trend of UF(k) is not significant at 95% confidence level (|U_0.05_| =± 1.96) between 1982 and 2008, except during the period of 2002–2004, and 2008.This result indicated that the annual global grassland scPDSI has a slight downward trend. Additionally, the intersection point of the UF(k) and UB(k) curves of the annual global grassland scPDSI occurred at 1996. Thus, the annual global grassland scPDSI mainly exhibited an increasing trend. In other words, the trend of global grassland drought has weakened in recent years.

### The characteristics of the annual global grassland scPDSI in different vegetation types

The grassland cover categories mainly include closed shrublands, open shrublands, woody savannas, savannas, and non-woody grasslands in this study. The change rate of the annual global grassland scPDSI is different in the five vegetation types (Table 1). The largest change rate of scPDSI occurred in woody savannas, while the lowest was in non-woody grasslands. The rank of the change rate of scPDSI in the five vegetation types was woody savannas (23.1%/10a, R^2^ = 0.3073) > savannas (22.2%/10a, R^2^ = 0.1993) > closed shrublands (9.3%/10a, R^2^ = 0.0745) > open shrublands (8.8%/10a, R^2^ = 0.0979) > non-herb grassland (1.3%/10a, R^2^ = 0.0024).

**Table 1:** The change rate of the annual global grassland scPDSI in different vegetation types.

Vegetation types	Change rate per decade (%)	R^2^
Closed Shrublands	9.3	0.0745
Open Shrublands	8.8	0.0979
Woody Savannas	23.1	0.3073
Savannas	22.2	0.1993
Non-woody Grasslands	1.3	0.0024

Spatial distribution of global grassland drought.

**Figure 2:**
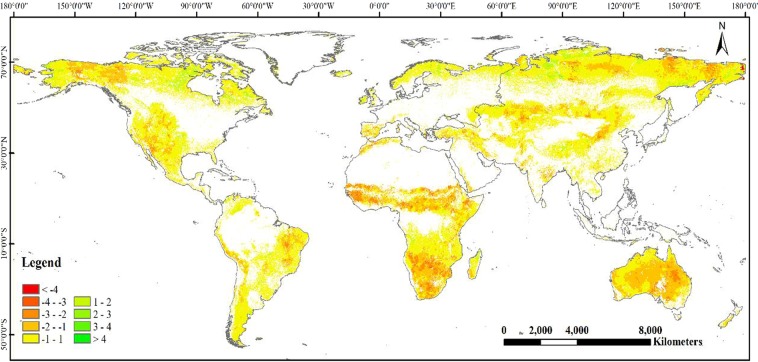
The mean scPDSI spatial distribution of global grassland during 1982–2008.

The scPDSI value is divided into four levels: mild drought (−1.99 to −1.00), moderate drought (−2.99 to −2.00), severe drought (−3.99 to −3.00), and extreme drought (below −4.00). Figure 2 shows that grassland drought affected area is up to 38.64% of the total grassland area. Additionally, the grassland area covered with mild drought accounts for 17.18% of the total grassland area, mainly concentrated in eastern Australia, central and southern parts of Africa, the Brazilian plateau of South America, and high latitudes of Canada and Russia. Similarity, the grassland area affected by moderate drought, severe drought, and extreme drought is 13.6%, 6.37% and 1.47% of the total grassland area, respectively, and chiefly distributed in eastern Australia, central and southern Africa.

**Figure 3:**
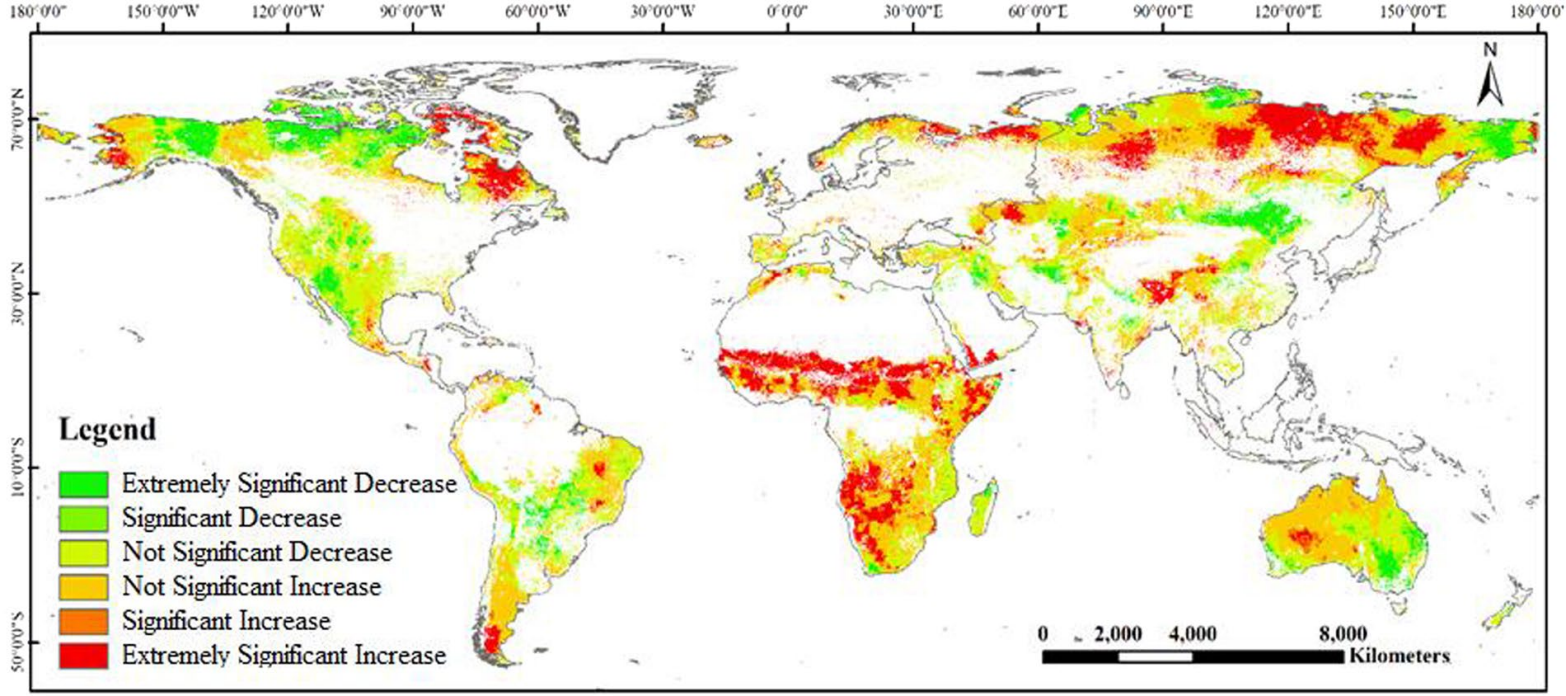
The variation trend of global grassland scPDSI index during 1982–2008.

Additionally, the variation trend of scPDSI is divided into four types: extremely significant increase, significant increase, significant decrease, and extremely significant decrease. Figure 3 shows the proportions of extremely significant increase, significant increase, significant decrease, and extremely significant decrease was 14.06%, 4.96%, 5.17% and 9.91%, respectively. In other words, greater than 19.02% of the global grassland area experienced an increasing trend, and mainly distributed in northeastern Russia, central and southern Africa, northeastern Canada, and Western Australia. However, 15.08% of the global grassland area is on a decline, and chiefly concentrated in northern North America, Brazil Plateau, Mongolia Plateau, and Eastern Australia.

### Correlations between scPDSI and the global grassland NPP

**Figure 4:**
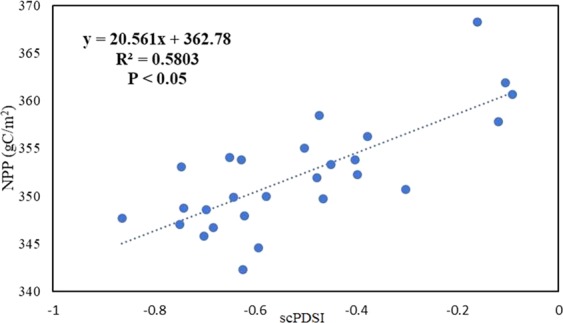
The correlation coefficient between annual NPP and scPDSI from 1982 to2008.

Figure 4 shows the correlations between scPDSI and global grassland NPP from 1982 to 2008. Quantitative relationship between scPDSI and global grassland NPP is established by linear fitting for each grid cell. The annual scPDSI has a positive significant with grassland NPP (P < 0.05, R^2^ = 0.58), suggesting that drought reduced grassland NPP during our study period.

**Figure 5:**
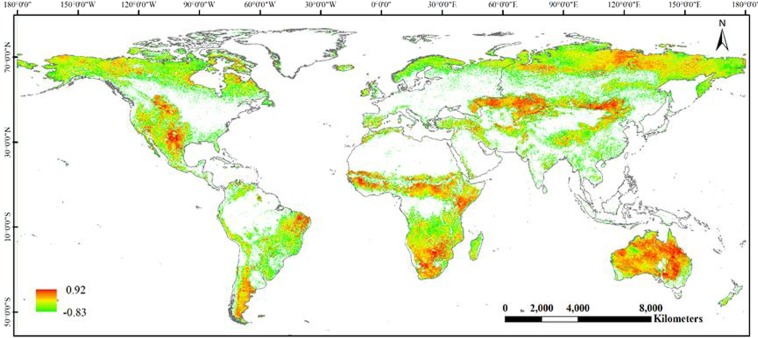
The spatial correlations between scPDSI and grassland NPP during 1982–2008.

Figure 5 shows spatial correlations between scPDSI and global grassland NPP during 1982–2008. It can be seen that the correlation coefficient is from −0.83 to 0.92. The correlation coefficient was divided into positive correlation coefficient (0–0.92) and negative correlation coefficient (−0.83–0). The region with positive correlation is mainly distributed in Kazakh grassland, Mongolian Plateau, central and southern Africa, and most of Australia. However, the region with negative correlation chiefly concentrated in high latitudes, Brazilian Highlands, Qinghai-Tibet Plateau, Katanga Plateau.

From the different grassland types, the correlation coefficients between scPDSI and grassland NPP of the Closed Shrublands, Open Shrublands, Woody Savannas, Savannas, and non-woody grassland was 0.15, 0.08, 0.03, 0.13, and 0.14, respectively. The order of the correlation coefficient between scPDSI and NPP for each grassland type was Closed Shrublands > Non-woody grassland > Savannas > Open Shrublands > Woody Savannas. The correlation coefficient between scPDSI and Closed Shrublands, non-woody grassland, and Savannas is relatively large, which indicates that the three types of grassland are more susceptible to drought.

**Figure 6:**
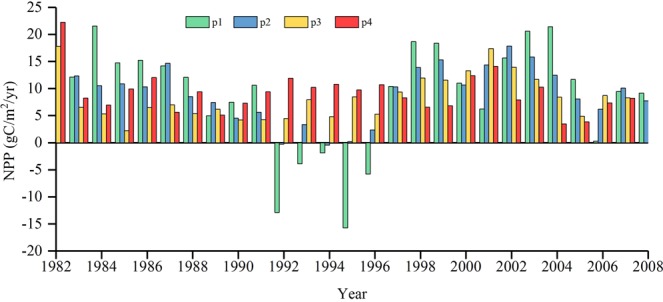
The change of annual mean grassland NPP at different drought levels during 1982–2008. p1, p2, p3, and p4 represent annual NPP changed at the mild drought, moderate drought, severe drought, and extreme drought, respectively.

Figure 6 illustrates the change of the annual mean grassland NPP at different drought levels during 1982–2008. The annual mean grassland NPP affected by droughts revealed a slight fluctuation during the study period. However, the annual mean grassland NPP affected by drought decreased at mild drought during from 1992 to 1996, with 12.87 gC/m^2^, 3.9 gC/m^2^, 1.85 gC/m^2^, 15.73 gC/m^2^, and 5.77 gC/m^2^ compared with the average of 1982–2008, respectively.

## Discussion

Understanding the impact of drought on grassland NPP is one of the basic objects of global change study^35^. Droughts had an important effect on the NPP, and this was confirmed by Zhao and Running (2010) and Pei *et al*.^12,25^. In this study, we assessed the impact of drought on grassland NPP at difference drought levels based on scPDSI during 1982 to 2008.

A previous study reported that severe drought had a much greater impact on regional NPP than mild drought^36^. However, we found that the annual mean grassland NPP decreased only under mild drought during 1992 to 1996. That is, some drought reduced grassland NPP, whereas the others did not. Additionally, the annual global grassland scPDSI mainly showed an increasing trend, suggesting that the global grassland drought alleviated to some extent that consistent with previously demonstrated^13^. Besides, the grassland affected area covered with mild drought accounts for 17.18% of the total. This also accords with our earlier observations, which showed that mild drought was the most important affecting factors in this study. The temperature and precipitation significantly contributed to annual grassland NPP variability^37^. Drought is mainly driven by precipitation and temperature; and NPP is the production through the process of photosynthesis. Therefore, drought stress aggravated photoinhibition of photosynthesis^38^, consequently affecting grassland NPP. Besides, drought on physio-ecological processes of plants and mechanism of drought resistance of plant is another explanation. There are, however, other possible explanations. Drought has the lag effects on grassland ecosystems, thus, grassland NPP falls behind of drought occurred. Previous-year drought controls a significant fraction of current-year production, and the magnitude of the response will increase with time^39^. The lagged effects of drought on vegetative growth is another explanation, which had been reported in a previous study^40^. Our result was also associated with the previous finding of temporal-spatial characteristics of drought events^41^ and El Niño events (1982 to1983, 1987 to 1988, and 1997 to1998)^25^.

We also quantified the contribution of drought to different type grassland NPP at the global scale. The results showed that the change rate of the annual global grassland scPDSI was difference in different grassland type. The order of the correlation coefficient between SCPDSI and NPP for each grassland type was: Closed Shrublands > Non-woody grassland > Savannas > Open Shrublands > Woody Savannas. The mainly reason was that the different grassland types had the different resistance and resilience of ecosystems to drought disturbances. Additionally, regional diversities of drought intensity, drought duration, and areal extents might one of the explanations. Moreover, drought intensities had a stronger correlation between droughts and NPP anomalies occurred during or after the time at which drought intensities reached their peak values^13^. In addition, the changes in grassland NPP in different grassland types are also related to other factors, such as vegetation difference index (NDVI), radiation and evaporation^42^. Besides, the different type of vegetation has the difference physiological regulation mechanism to drought and lag responses of vegetation to the precipitation deficits may be another reason.

## Conclusions

We have analyzed the impact of drought on grassland NPP at global scale based on scPDSI during 1982 to 2008. The results showed that drought had a significant influence on grassland NPP. The overall change trend of scPDSI showed an increasing trend and the correlation coefficient was from −0.83 to 0.92. We also found that the annual mean grassland NPP decreased only under mild drought during 1992 to 1996, suggesting that some droughts reduced the grassland NPP, whereas the others did not.

The change rate of the annual global grassland scPDSI was difference in different grassland type. The order of the correlation coefficient between SCPDSI and NPP for each grassland type was: Closed Shrublands > Non-woody grassland > Savannas > Open Shrublands > Woody Savannas. The result indicated that the different grassland types had the different resistance and resilience of ecosystems to drought disturbances due to their difference physiological regulation mechanism.

In present study, we only analyzed the impacts of drought on grassland NPP at different drought level. Future research should consider the potential effects of drought more carefully, for example human activities, wildfires, overgrazing, pests, and other factors.

## Data and Methods

### NPP data set

NPP estimation based on productivity efficiency approach was first introduced by Monteith (1972)^43^. Vegetation NPP can be estimated using the variables of the photosynthetically active radiation absorbed by green vegetation (APAR) and the efficiency by which that radiation is converted to plant biomass increment^44,45^. Several types of models have been developed to estimate NPP at large scales^44^. Previous studies showed that Moderate Resolution Imaging Spectroradiometer (MODIS) data can be used to estimate NPP based on Carnegie-Ames-Stanford Approach (CASA) model^46^. Thus, we estimated the global grasslad NPP from 1982 to 2008 by using CASA model. We selected a long time series of NDVI data set from 1982 to 2008 from the web site at http://ladsweb.nascom.nasa.gov/data/search.html. All of the related databases were resized to 1-km spatial resolution. The NPP estimation and CASA model have been decribed in more detail in prevous stdudies^44,47^.

### Global land cover data set

The global land cover data was from the MOD12Q1 product (http: //modis-land.gsfc.nasa.gov/landcover.html/). The classes are defined according to the International Geosphere-Biosphere Project (IGBP) land cover system based on satellite imagery of land cover and vegetation type^48^. In this study, the grassland cover categories mainly include closed shrublands, open shrublands, woody savannas, savannas, and non-woody grasslands^10,49^ (Table 2). All files of land cover data were merged together and converted into TIFF format using the MODIS reprojection tool, and then converted into grid format to match the NPP data.

**Table 2:** The description of the grassland types in IGBP class scheme.

Grassland types	Description
Closed Shrublands	Lands with woody vegetation with a height less than 2 meters. The total percent cover, including the herbaceous understory, exceeds 60%. The shrub foliage can be either evergreen or deciduous.
Open Shrublands	Lands with woody vegetation with a height less than 2 meters, and sparse herbaceous understory. Total percent cover is less than 60%. The shrub foliage can be either evergreen or deciduous.
Woody Savannas	Lands with and herbaceous understory, typically graminoids, and with tree and shrub cover between 30 and 60%. The tree and shrub cover height exceeds 2 meters.
Savannas	Lands with an herbaceous understory, typically graminoids, and with tree and shrub cover between 10 and 30%. The tree and shrub cover height exceeds 2 meters.
Non-woodyGrasslands	Lands with herbaceous types of cover, typically graminoids. Tree and shrub cover is less than 10%.

### Drought disaster data set

The self-calibrated Palmer Drought Severity Index (scPDSI^50^) is a modification of the original measure of regional moisture availability that better allows comparison of drought from different regions^51^. Recently, an enhanced version of the global grid monthly scPDSI dataset was released for the period 1901–2009^52^. It was widely used as the basis for investigating long-term changes in drought severity^53^. Therefore, we selected apart of scPDSI dataset (1982–2008) to qualify the drought at the global scale. The scPDSI is available at http://www.cru.uea.ac.uk.

### Descripetion of self-calibrating PDSI (scPDSI)

Palmer drought severity index (PDSI) is a widely used drought index since 1965^54^. It is calculated based on temperature and precipitation information. Since the behavior of the index at various locations is not consistent, it is difficult to make spatial comparisons of PDSI. Thus, the self-calibrating PDSI (scPDSI) proposed by Wells *et al*.^55^. The scPDSI significantly improved PDSI comparability to each location and more reasonable for monitoring extreme wet and dry events. The scPDSI automatically calibrates the behavior of the index at any position by replacing empirical constants in the index computation with dynamically calculated values^52^.

The scPDSI reduces the excessive frequency of extreme events, when compared to the original PDSI. To quantitatively assess the drought, the scPDSI was selected among the many drought indices. In this study, we used the scPDSI to analyze the temporal changes in the potential drought impacts under climate change. The drought indices classification is shown in Table 3. The scPDSI values of –1.99 to –1.0, –2.99 to –2.0, –3.99 to –3.0, and less than –4.0 represent mild drought, moderate drought, severe drought, and extreme drought, respectively^56^.

**Table 3:** The scPDSI drought category classification.

scPDSI value Class	Situation classification
−0.99 to 0.99	Normal or wet spell
−1.99 to −1.00	Mild drought
−2.99 to −2.00	Moderate drought
−3.99 to −3.00	Severe drought
Below −4.00	Extreme drought

Simple linear regression was employed to analyze the annual variation of the global grassland drought dynamics during the study period. The slope of the trend line in the multiyear regression equation for a single pixel represents the inter-annual variation rate, which is solved by the ordinary least-squares method. Slope shows positive, suggesting that the grassland drought has an increasing trend. Whereas slope is negative, indicating that the grassland drought has an decreasing trend^57^.

The significance of the variation tendency was determined by using the statistic F-test to represent the confidence level of variation in our study. Through the significance test (P < 0.01 or P < 0.05), the correlation coefficient can indicate whether the trend is “extreme significant” or “significant”. The significance levels of F were classified into six levels: extremely significant decrease(ESD, F_slope_ < 0, P < 0.01), significant decrease (SD, F_slope_ < 0, 0.01 < P < 0.05), non-significant decrease (NSD, F_slope_ < 0, P > 0.05), non-significant increase (NSI, F_slope_ > 0, P > 0.05), significant increase (SI,F_slope_ > 0, 0.01 < P < 0.05) and extremely significant increase (ESI, F_slope_ > 0, P < 0.01).

The nonparametric Mann–Kendall method is employed to detect possible trends of drought indices^58,59^. The results of the M–K test are heavily affected by serial correlation. Thus, we adopt the Yue and Pilon method to remove the serial correlation^60^. The self-calibrating Palmer Drought Severity Index (scPDSI) have been calculated for the period 1901–2009 based on the CRU TS 3.10.01 data sets and employs the original severity scale^61^.
